# Depth-dependent influence of biochar application on the abundance and community structure of diazotrophic under sugarcane growth

**DOI:** 10.1371/journal.pone.0253970

**Published:** 2021-07-19

**Authors:** Nyumah Fallah, Ziqi Yang, Muhammad Tayyab, Caifang Zhang, Ahmad Yusuf Abubakar, Zhaoli Lin, Ziqin Pang, Americ Allison, Hua Zhang

**Affiliations:** 1 Key Laboratory of Sugarcane Biology and Genetic Breeding, Ministry of Agriculture, Fujian Agriculture and Forestry University, Fuzhou, 350002, China; 2 College of Agriculture, Fujian Agriculture and Forestry University, Fuzhou, 350002, China; Government College University Faisalabad, Pakistan, PAKISTAN

## Abstract

Despite progress in understanding diazotrophic distribution in surface soils, few studies have investigated the distribution of diazotrophic bacteria in deeper soil layers. Here, we leveraged high-throughput sequencing (HTS) of nifH genes obtained to assess the influence of biochar amended soil (BC) and control (CK), and soil depths (0–20, 20–40 and 40–60 cm) on diazotrophic abundance and community structures, soil enzyme activities and physio-chemical properties. Multivariate ANOVA analysis revealed that soil depth had profound impact on majority of the soil parameters measured than fertilization. Although soil physio-chemical properties, enzymes activities, diazotrophic genera and enriched operational taxonomic units (OTUs) were significantly influenced across the entire soil profiles, we also observed that BC amended soil significantly increased cane stalk height and weight, nitrate (NO_3_^-^), ammonium (NH_4_^+^), organic matter (OM), total carbon (TC) and available potassium (AK), and enhanced diazotrophic genera in soil depth 0–20 cm compared to CK treatment. Soil TC, total nitrogen (TN), OM and NH_4_^+^ were the major impact factors shifting diazotrophic community structures in soil depth 0–20 cm. Overall, these results were more pronounced in 0–20 cm soil depth in BC than CK treatment.

## 1. Introduction

Sugarcane (*Saccharum officinarum* L.) is an economically vital crop planted in tropical and subtropical regions globally with an annual production of approximately 16 million tons. It contributes considerably to the sugar and biofuel-producing industries [1–3]. Fertilization is a crucial agricultural approach that not only improves plant nutrient storage but also simultaneously alters soil attributes and microbial communities [4,5]. In the past few decades, extensive fertilization, especially nitrogen (N) fertilizer, has been used to raise sugarcane production to meet the growing sugar demand [5,6]. Although inorganic fertilization has a positive effect on sugarcane yield, on the other hand, it has unfavorable indirect effects on soil quality by causing soil acidification, enhancing soil pathogens, intensifying nitrification and leaching of nitrates [7–9]. In contrast, organic fertilization is an alternative approach to chemical fertilization to mitigate soil acidification and improve soil nutrient status, thus ensuring sugarcane productivity [2].

Biochar (BC) a soil supplement that is derived from pyrolysis of organic materials at a higher temperature under a limited oxygen environment. It is a stable organic material, dark in color, porous and carbonaceous [10,11]. Biochar as a soil amendment is a promising approach to improve soil fertility because it plays an important role in establishing reliable carbon storage [12]. Moreover, BC amendment facilitates environmental functions, and mitigating greenhouse gas emissions [13,14]. In addition, adding BC to the soil can increase nutrient retention by absorbing carbon and reducing soil acidity [11]. Biochar induced changes may influence not only nutrients and organic materials cycling, but also plant growth and development [10]. Studies have shown that BC amended soil caused changes in soil bacterial abundance and community composition [14,15]. However, our understanding of the BC amendment on soil fertility, soil bacterial community composition and their activities with a focus on soil depth as well as overall impact on sugarcane growth is limited.

Soil microbial community plays a pivotal role in nutrients recycling, by changing inorganic compounds and decaying organic materials [16,17], and maintaining quality underground water, thereby improving environmental functions [18]. Soil profiles serve as an ecological filter for many soil microorganisms [19]. Furthermore, the existence of the microbial web in soil is very complex, as well as their compositions and diversity differ in soil layer [20]. Soil microbial are essential in N cycling. They modulate soil available N that are required by plants [16].

Biological nitrogen fixation (BNF) is an important N source which provides nutrient for crops [20,21]. Diazotrophic (N2 fixers) are diverse group of soil bacteria that are capable of transforming atmospheric N2 to plant-available ammonium (NH_4_^+^) [22], by nitrogenase, a universal enzyme [23,24]. This process serves as a major source of N, accounting for about 97% into the environment [25]. Recently, a molecular marker of nifH gene family which encodes for the nitrogenase reductase subunit has been used to analyze diazotrophic community structures [25,26]. N2 fixer is sensitive to soil amendment practices. For instance, short-term application of biochar increased diazotrophic abundance and changed community compositions and increased the biological nitrogen fixation rate in an alkaline soil under soybean cultivation, and revealed the highest abundance of nifH gene [14]. Diazotrophic abundance and community structures also decrease with increasing soil depth [27]. Jichen et al. [23] reported that soil depth was the main indicator that altered diazotrophic structures rather than fertilization or sampling time. In addition, Reardon et al. [25] documented that soil depth was the influencing factor that altered nifH gene abundance which was less in 10–20 cm as compared to 0–10 cm soil layer. However, our knowledge of the distribution pattern of soil microbe community structure and their related activities have been limited to topsoil 0–15 cm [19], where soil bacteria and organic matter is abundant [28]. Moreover, not much is known about diazotrophic relative abundance and community structure, as well as their relationship with soil environmental variables in deeper soil layer under biochar application during sugarcane growth. To address these knowledge gaps, a field experiment was designed using biochar as soil amendments during sugarcane cultivation to assess the effect on diazotrophic, soil biochemical properties and sugarcane parameters. The study sought to: (a) investigate the variation in diazotrophic abundance and community structures in three soil profiles (0–20, 20–40 and 40–60 cm) under biochar application; (b) explore the relationships between the diazotrophic community structures and environmental variables using high-throughput sequencing (HTS).

## 2. Materials and methods

A field experiment was conducted in March 2019 at the Sugarcane Research Center of Fujian Agriculture and Forestry University in Fuzhou, Fujian Province (latitude 26°5′0′′ east longitude 119°13′47′′). The local climate of the area is subtropical monsoon with an annual average temperature of 20°C and a rainfall of 1369 mm. The experiment was set in a randomized block design with two different treatments and each treatment was replicated three times. Each replicate covered an area of 25 m^2^ (5 m x 5 m). The two treatments used were: control (CK) and biochar amended soil (BC). Biochar was applied at the rate of 20 t ha-^1^ on March 15, 2019. The biochar used in this study was bought from Nanjing Qinfeng Crop Straw Technology Company, China. The biochar was produced from sugarcane straw at the 550–650°C. The properties of the biochar were: TC = 09.08 mg kg^-1^; TN = 1.27 mg kg^-1^; content pH (H_2_O) = 11.01; AP = 5.21 mg kg^-1^ and AK = 17.03 mg kg^-1^. While TC = 13.22 mg kg-^1^, TN = 19.54 mg kg-^1^, pH = 8.89, AK = 14.01 mg kg-^1^, AP = 9.11 mg kg-^1^, OM = 10.22 mg kg-^1^, EC = 3.11 dS m-^1^, NO_3_^-^ = 8.76 mg kg-^1^ and NH_4_^+^ = 11.02 mg kg-^1^ were measured for the initial soil properties. The biochar was uniformly spread to the soil surface and instantly mixed into the plowed soil at the depth of 0–60 cm before cultivating the sugarcane. Sugarcane stems were cut at about 10–12 cm in length, maintaining two buds on each sett [29], and 10 setts were planted on each row with 0.3 m plant-to-plant spacing and 0.5 m row-to-row spacing.

### 2.1 Plant cane growth parameters

Sugarcane height was measured in centimeter (cm) with the help of a meter rod from the soil surface to the top of the sugarcane. Mean plant height was calculated by taking the average of three replications. Cane plant fresh weights were used to determine each stalk weight (kg stalk-^1^). Twenty sugarcane stalks were randomly sampled from each row, milled and analyzed the sucrose content using the method described by Jackson and Jayanthy [30].

### 2.2 Soil sampling

The soil samples were collected from different depths (0–20, 20–40 and 40–60 cm) in October of 2020. For every plot, a sample was collected at five different spots, homogenized and mixed accordingly forming one sample. Sieving of soils was done using 2-mm mesh. Plant residues and stones were removed. Portion of the soil was stored at -20°C for the extraction of DNA and enzyme activities. The remaining soil samples were stored at 4°C for analyzing soil environmental variables.

### 2.3 Analysis of soil physio-chemical properties

Air-dried soils were used to analyze soil pH, available phosphorus (AP), available potassium (AK), total carbon (TC), total nitrogen (TN) and organic matter (OM). Soil pH was determined using glass electrode pH meter (water/dry soil, 2.5:1) [31]. Available phosphorus was estimated using the Molybdenum Blue method [32]. The Flash Smart elemental analyzer (Thermo ScientificTM, Waltham, MA, USA) was used to measure the TN and TC. Soil AK was extracted by ammonium acetate and determined by atomic absorption spectrophotometry [32]. The Walkley-black method was adopted measure soil OM, which consisted of the soil organic matter oxidation by K2Cr2O7 and H2SO4, and FeSO4 was then used for titration [33]. A fresh soil sample was used for the extraction of ammonium (NH_4_^+^) and nitrate (NO_3_^-^) as described by Sun et al. [34].

### 2.4 Measurement of soil enzyme activities

Soil enzyme activities were determined as described by Sun et al. [35] and Tayyab et al. [36]. Incubation of soil was carried out using buffer sodium carboxymethylcellulose solution, cellulose (glucose, mg/g 24 h, 37°C) activity was measured colorimetrically by quantifying a decrease in 3,5-dinitrosalicylic acid from reducing sugar. Soil urea activity (NH_3_-N, mg/g 24 h, 37°C) was measured using improved sodium phenolate and sodium hypochlorite colorimetry. While a nitrophenyl phosphate disodium substrate was used to determine acid phosphatase activity (phenol, ug/g, 1 h, 37°C). After buffering the soil with p-nitrophenyl-β-glucopyranoside, the β-glucosidase activity (p-nitrophenyl, ug/g, 1 h, 37°C) was determined using a colorimeteric p-nitrophenol assay.

### 2.5 Soil DNA extraction, amplification, sequencing, and data processing

The extraction of soil metagenomics DNA from 0.5 g fresh soil was carried out using Fast DNATM Spin Kit (MP Biomedicals, LLC, Santa Ana, USA) following the manufacturer guidelines. Assessment of DNA quality and quantity were carried out by calculating their absorbance (A260 and 280nm) using a spectrophotometer. The extracts were then stored at -20°C awaiting sequencing.

High throughput sequencing was used to determine diazotrophic community structures using the Illumina Miseq platform. The nifH amplification was carried out using primer pair PolF and PolR [37] combined with Illumina adaptor sequences and barcode sequences [38]. Sample libraries were generated from the purified PCR products. The Miseq 300 cycle Kit was used for paired-end sequencing on a Miseq benchtop sequencer (Illumina, San Diego, CA, United States). The raw nifH gene sequences were separated using sample based on their barcodes and permitting up to one mismatch. Btrim [39] was used to do quality trimming. Forward and reverse reads were merged into full-length sequences using FLASH [40], and sequences with short or contained ambiguous bases were removed. Resampling was done with 10,000 sequences per sample randomly. UCLUST was used to classify the operational taxonomic units (OTUs) at the 97% similarity level, and singletons were removed. The frameshift caused by insertions and deletion in DNA sequences were checked and corrected by RDP FrameBox. Valid nifH gene sequences (300–320 pb) were translated in proteins sequences and taxonomic assignment was performed using RDP FrameBox tool [41], after the processing.

### 2.6 Statistical analysis

A nonmetric multidimensional scaling (NMDS) analysis was employed to assess the differences in diazotrophic community structures in different soil depths. Bray-Curtis dissimilarities were used to analyze similarities (ANOSIM) to estimate the dissimilarity in diazotrophic community structures in 0–20, 20–40 and 40–60 cm depths. Redundancy analysis (RDA) was further performed in the different soil profiles to determine the relationship between diazotrophic community structures and environmental variables. Venn diagrams were plotted to visualize unique and overlap diazotrophic genera in different soil depths (http://bioinfogp.cnb.csic.es/tools/venny/index.html). Ternary plot analysis was then conducted using R language based package ggtern an extension of package ggplot2 to determine the enriched abundant OTUs of diazotrophic bacteria in different soil depths of the combined samples. Pearson’s correlation coefficients were employed separately for the different soil depths to test the relationship between soil properties and diazotrophic community structures at both the phyla and genera levels, using R-software [42]. The effects of soil depth and fertilization regime and their association with different soil parameters relating to diazotrophic bacteria, soil enzyme activities and soil physiochemical were performed with multivariate ANOVA in the 0–20 cm, 20–40 cm and 40–60 cm soil depths. Due to the significance of the interactions (*p* < 0.05), the Tukey’s HSD post-hoc analysis was performed.

## 3. Results

### 3.1 Response of sugarcane parameters to biochar amended soil

**Fig 1:**
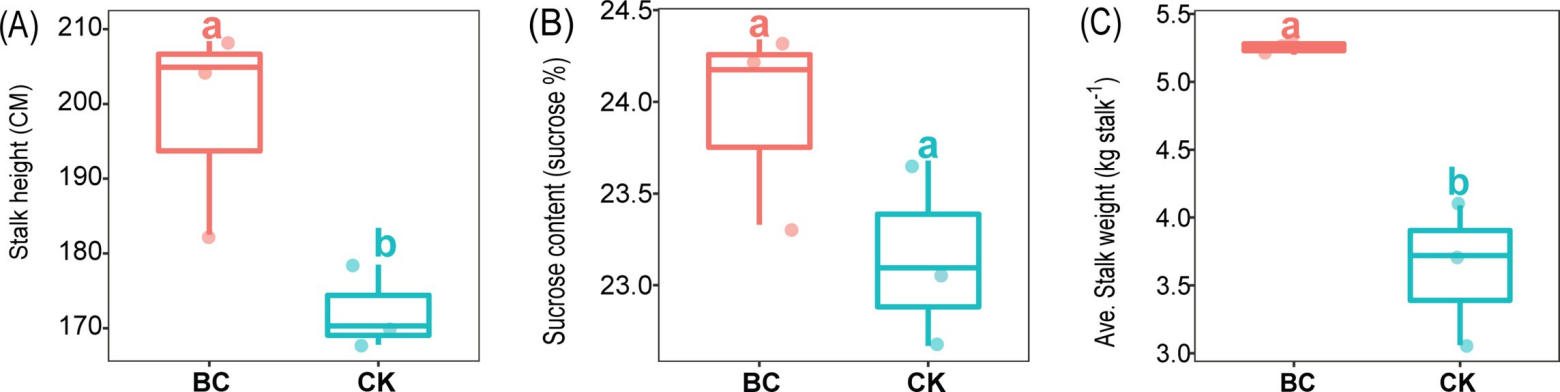
Impact of biochar amendment on sugarcane agronomic parameters. Boxplots with various lowercase letters indicate significant (*p <* 0.05) differences between treatments based on Tukey’s HSD test. CK: Fertilization with N, P and K; BC: Fertilization with biochar.

Biochar amended soil significantly increased (*P <* 0.05) sugarcane stalk number, stalk weight compared with CK treatment (Fig 1A and 1C). However, compared to CK treatment, BC amended soil had no impact on sucrose content (Fig 1B).

### 3.2 Soil environmental variables

**Fig 2:**
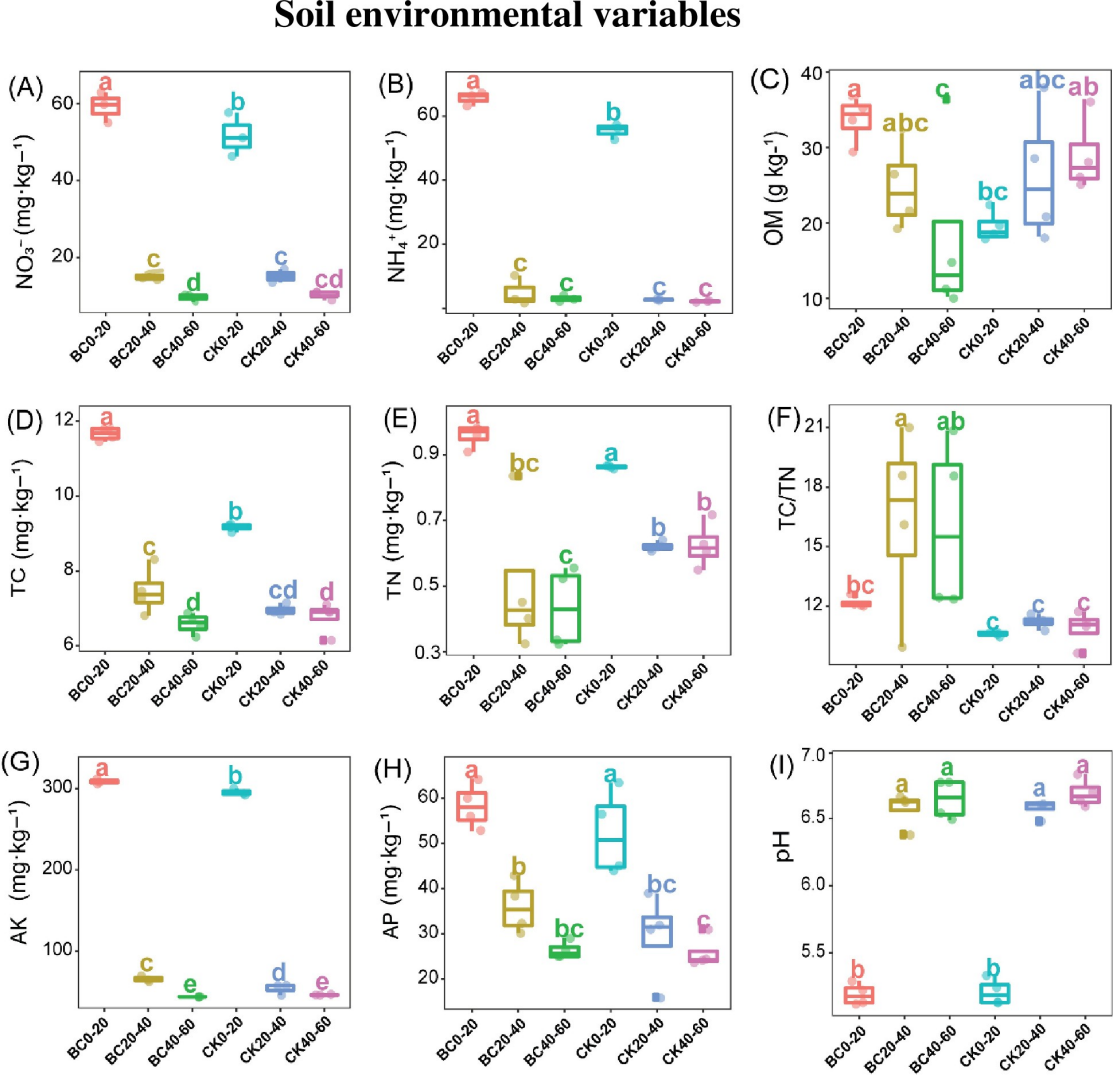
Soil environmental variables at three soil layers under biochar (BC) amended soil compared to control (CK). Boxplots with different lowercase letters depict significant differences between treatments (Tukey test, *p* < 0.05). (A) NH_4_^+^, ammonium; (B) NO_3_-, nitrate; (C) OM, soil organic matter; (D) TC, total soil carbon; (E) TN, total nitrogen; (F) TC/TN, carbon/nitrogen ratio; (G) AK, available potassium; (H) AP, available phosphorus; (I) pH potential hydrogen.

**Fig 3:**
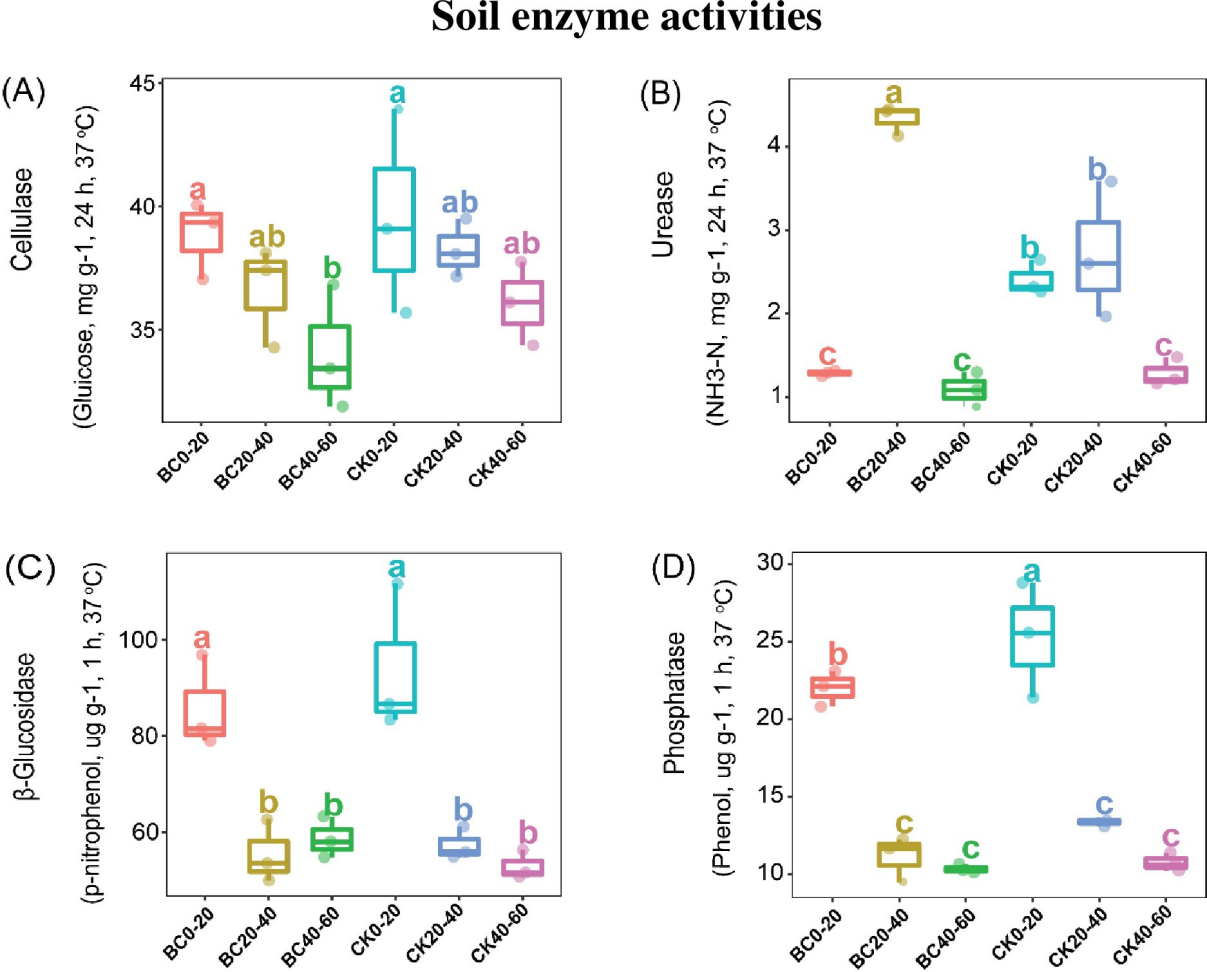
Soil enzymatic activities in different soil profiles. (A) cellulase activity, (B) urease activity, (C) β-Glucosidase, (D) acid phosphatase activity under BC, biochar amendment; compared to the CK, control at different soil depths. Boxplots with various lowercase letters indicate a significant difference between treatments based on Tukey’s HSD test (*p* < 0.05).

Soil physio-chemical properties varied considerable with increasing soil depth, and soil admendmets practices (Fig 2). Soil NO_3_^-^, NH_4_^+^, TC, AP, AK and TN significantly decreased with soil depth in all the treatments (Fig 2A, 2B, 2D, 2E, 2G and 2H). Moreover, soil OM decreased with increasing soil depth in BC treatment, while in CK, OM fluctuated (Fig 2C). In BC treatment, TC/TN fluctuated across the entire soil depth, but revealed no change in entire soil profile in CK. Soil pH in both CK and BC were significantly higher (*p* < 0.05) in 20–60 cm than 0–20 cm soil depth (Fig 2I). Regarding various treatments, BC amended soil significantly improved (*p* < 0.05) soil NO_3_^-^, NH_4_^+^, OM, TC and AK in one soil depth (0–20 cm) (Fig 2A–2D and 2G) compared to CK. Moreover, BC treatment significantly enhanced (*p* < 0.05) TC/TN and AK at soil depths (20–40, 40–60 cm, and 0–20 and 20–40 cm, respectively) compared with CK treatment (Figs 2F and 3G). However, in soil depth (40–60 cm) soil TN significantly diminished under BC amended soil compared with CK treatment (Fig 2E). In the whole soil profile (0–60 cm), BC treatment did not change soil pH in comparison to that under CK treatment (Fig 2I).

### 3.3 Soil enzyme activities

In both CK and BC treatments, cellulose decreased with soil depth (Fig 3A). Acid phosphatase activity and β-glucosidase activity were significantly higher (*p* < 0.05) in topsoil (0–20 cm) than soil depth 20–60 cm in both CK and BC treatments (Fig 3C and 3D). Additionally, urease activity was significantly higher (*p* < 0.05) in soil layer 20–40 cm than 0–20 and 40–60 cm soil profiles in BC treatment. Whereas in 0–40 cm soil layer, urease activity was stable compared to 40–60 cm soil profile in CK (Fig 3B). With respect the different treatments, cellulase and β-glucosidase activities in BC amended soil revealed no significant change in the entire soil layer (0–60 cm) compared to CK (Fig 3A and 3C). In soil layer (20–40 cm), urease activity increased significantly (*p* < 0.05) under BC treatment compared with CK. While in one soil depth (0–20 cm), urease activity was significantly higher (*p* < 0.05) in CK treatment (Fig 3B). In CK treatment, acid phosphatase activity was significantly higher (*p* < 0.05) in soil depth (0–20 cm) than BC amended soil but revealed no change in 20–60 cm under both treatments (Fig 3D).

### 3.4 Relative abundance of dominant diazotrophic phyla and genera

**Fig 4:**
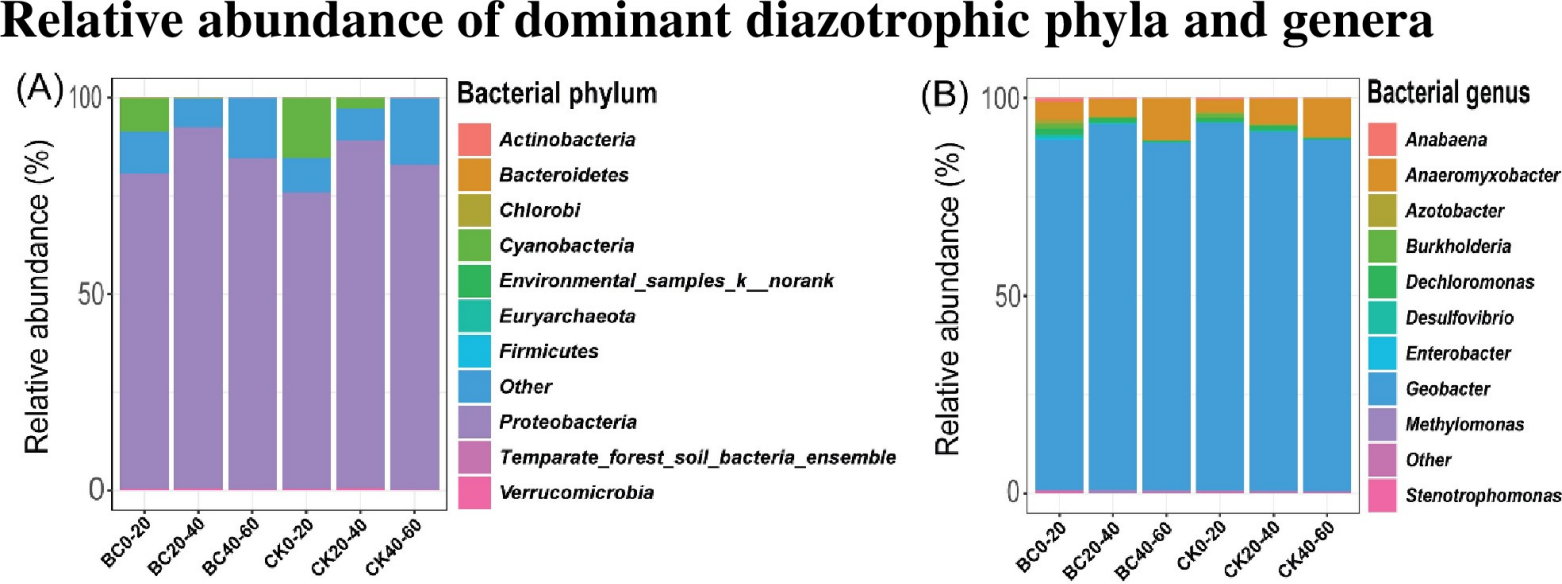
Diazotrophic bacterial relative abundance. (A) diazotrophic phyla, (B) diazotrophic genera under and biochar amended soil and control in different soil depths. “Other” indicates those identified phyla and genera that were beyond the top 10 phyla and genera.

The relative abundance of dominant diazotrophic phyla and genera levels were examined under BC and CK treatments at three different soil depths (0–20, 20–40 and 40–60 cm) (Fig 4). Our result revealed that soil depth and various soil amendment practices did not significantly alter diazotrophic phyla across the entire soil profiles (Fig 4A). However, *Proteobacteria* (80.2–92.0%) was the highly dominant diazotrophic phylum followed by *Cyanobacteria* (0.0–8.6%) and *Verrucomicrobia* (0.2–0.6%) in both CK and BC treatments (S1C, S1G and S1H Fig). Diazotrophic genera significantly changed with soil depth. At the genera level of diazotrophic, the relative abundance of *Geobacter* (88.6–93.6%) was highly dominant, followed by *Anaeromyxobacter* (4.5–10.8%), *Dechloromonas* (0.4–1.4%), *Burkholderia* (0.1–1.3%), *Desulfovibrio* (0.2–1.0%), *Methylomonas* (0.1–0.8%) and *Azotobacter* (0.0–1.0%) in all the samples (Fig 4B). However, *Anabaena* improved considerably (*p* < 0.05) in both CK and BC in 0–20 cm soil layer (S2A Fig). Whereas *Azotobacter*, *Burkholderia*, *Dechloromonas*, and *Enterobacter* significantly increased (*p* < 0.05) in BC treatment compared to 20–60 cm soil profile (S2B, S2C, S2G and S2J Fig). While in CK, genus *Azotobacter* revealed no change in the entire soil layer (S2B Fig). With regard to various treatments, BC amended soil improved genus *Anabaena*, *Burkholderia*, *Stenotrophomonas*, *Dechloromonas*, *Methylomonas* and *Enterobacter* relative abundance in one soil layers (0–20 cm and 20–40 cm) compared to CK, respectively (S2A, S2C, S2F, S2G, S2H and S2J Fig). However, compared to CK, BC amendment significantly diminished *Anaeromyxobacter* relative abundance in one soil layer (0–20 cm) (S2I Fig). Whereas *Geobacter* fluctuated in the entire soil profile in both CK and BC (S2E Fig).

### 3.5 Bacterial beta diversity

**Fig 5:**
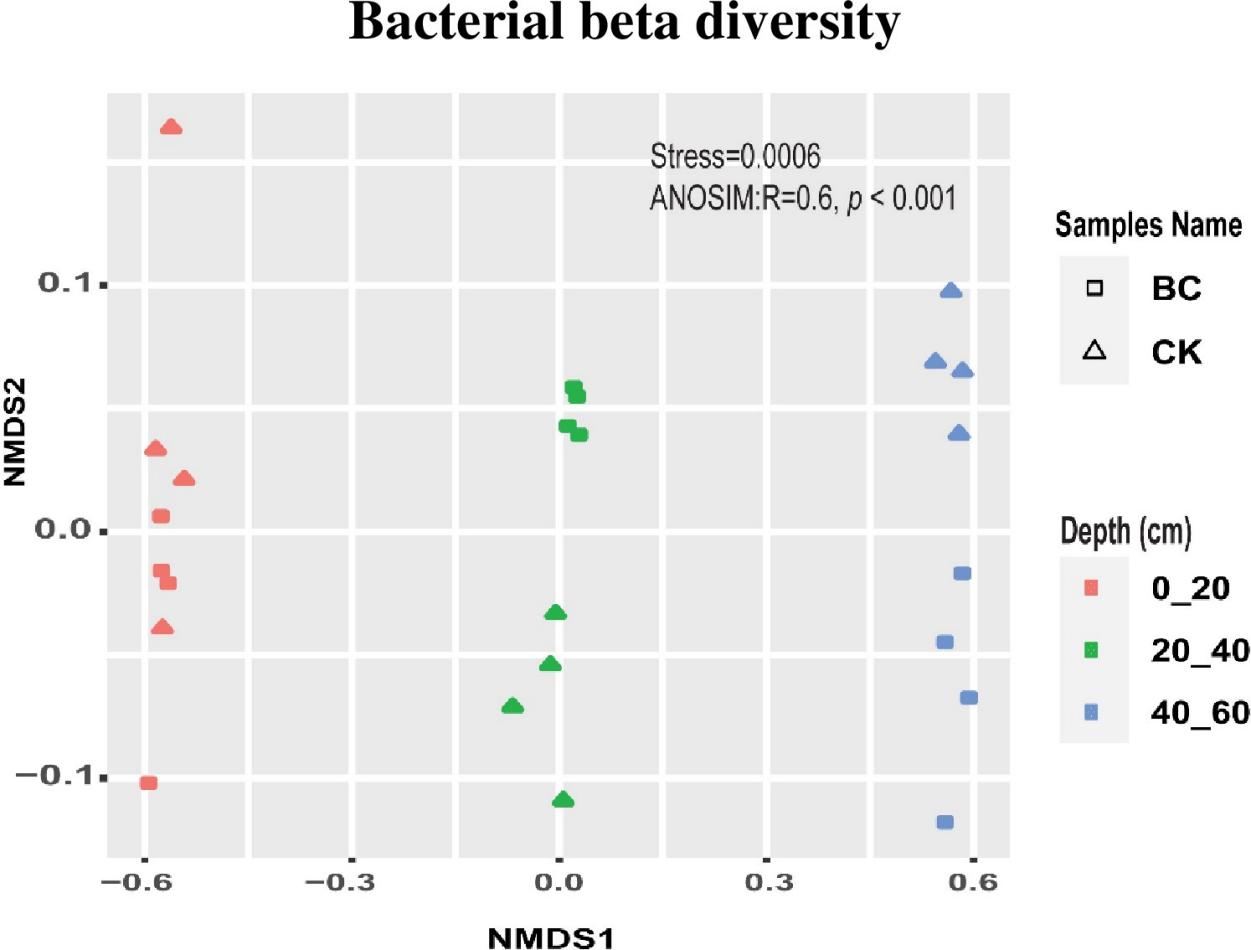
Analysis of nonparametric multidimensional scaling (NMDS) of the diazotrophic community structure in different soil depth. CK, control; BC, biochar amended soil.

A nonmetric multidimensional scaling (NMDS) analysis employed clearly revealed the separation of the different soil depths (0–20, 20–40 and 40–60 cm) (Fig 5). Moreover, an analysis of similarities (ANOSIM) reinforced further the significant differences (*R*^*2*^ *=* 0.56, *p <* 0.001) among the different soil depths.

### 3.6 Redundancy analysis

**Fig 6:**
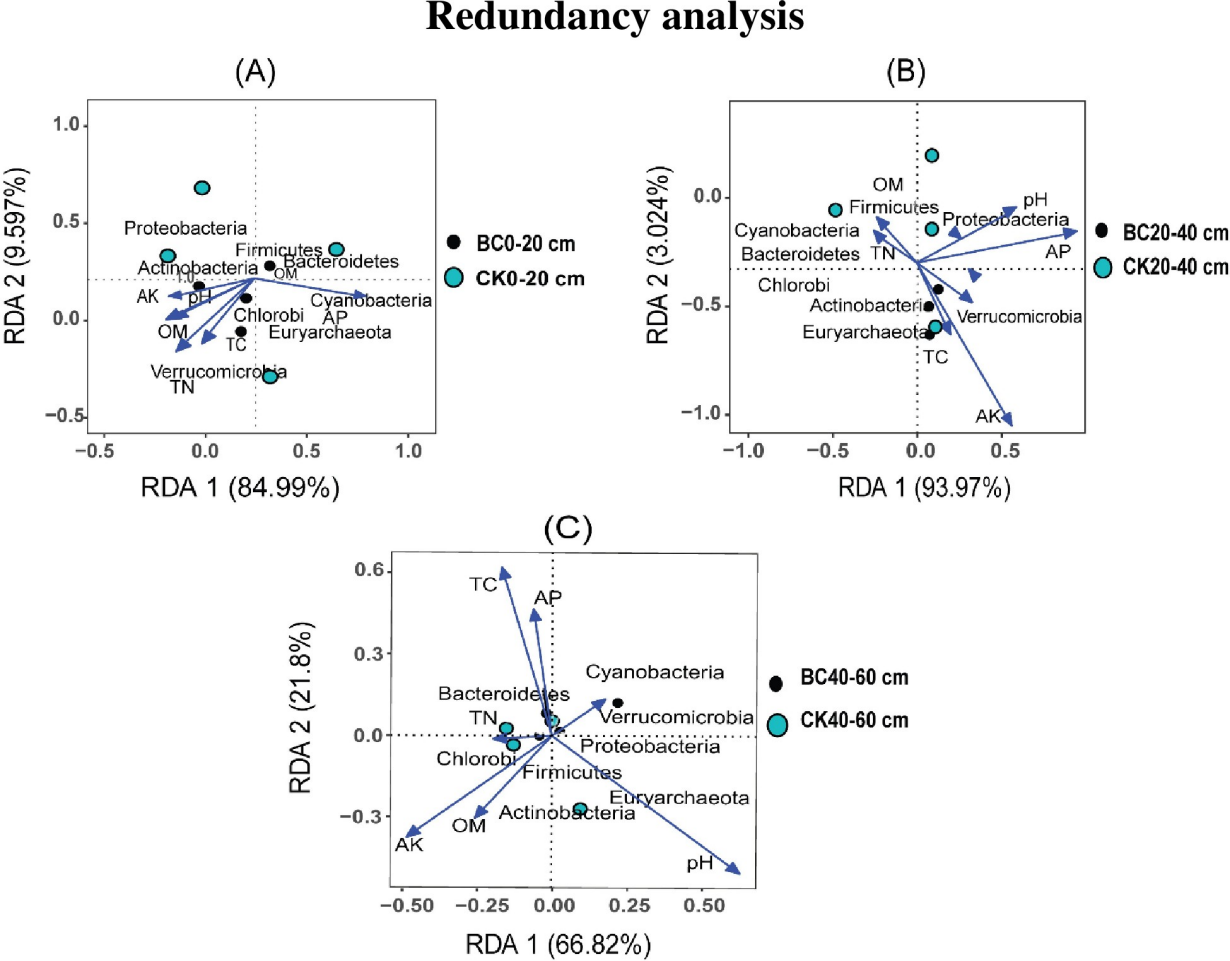
Redundancy analysis (RDA) of diazotrophic community composition and soil environmental variables. (A) diazotrophic phyla, (B) diazotrophic genera under and biochar amended soil and control in different soil depths.

From the results obtained from the NMDS analysis, a redundancy analysis (RDA) was performed in different soil depths to assess the effect of soil environmental variables on diazotrophic community structure (Fig 6). The analysis revealed that soil TC (*R*^*2*^ = 1.1024, *p <* 0.001), TN (*R*^*2*^ = 1.0984, *p <* 0.001), OM (*R*^*2*^ = 1.0872, *p<*0.001) and NH_4_^+^ (*R*^*2*^ = 1.0792, *p <* 0.001) were the minor impact factors that caused a shift in diazotrophic communities structure in soil depth 0–20 cm (Fig 6A). At soil depths 20–40 and 40–60 cm, soil AK (*R*^*2*^ = 1.2679, *p <* 0.001) and AK (*R*^*2*^ = 1.2168, *p <* 0.001) was the main driver shifting diazotrophic community structure, respectively (Fig 6B and 6C).

### 3.7 Bacterial alpha diversity

Diazotrophic community alpha diversity was analyzed accordingly in every sample along with various soil depths using an estimator of diversity. We observed that N2-fixers alpha diversity under BC amended soil did not vary considerably compared to CK. However, regarding soil layers, N-fixation bacterial alpha diversity revealed little change with soil depth in CK and BC treatments. (S1 and S2 Tables).

### 3.8 Diazotrophic distribution patterns in different soil depths

**Fig 7:**
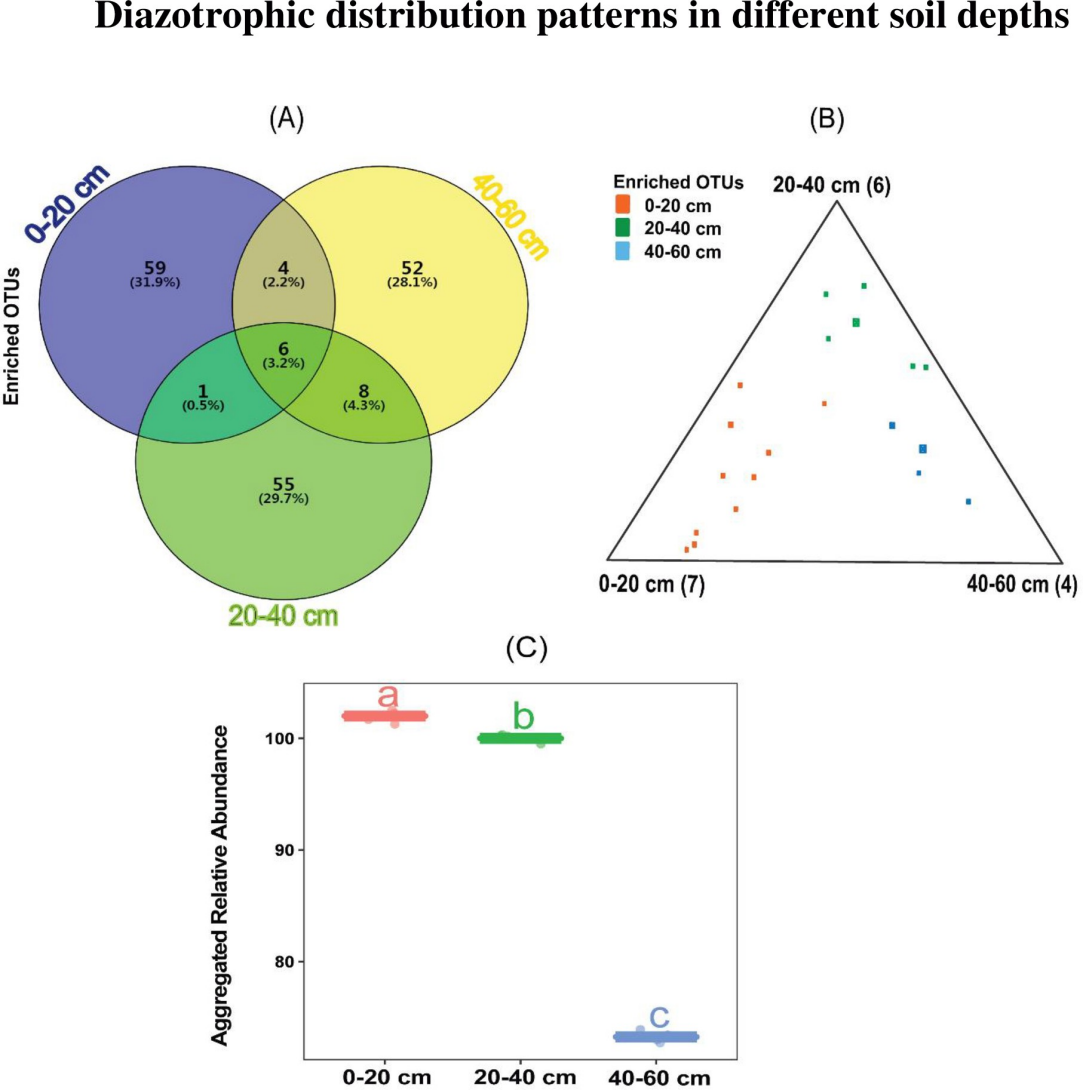
Diazotrophic genera enriched OTUs distribution patterns in different soil depths, A-C. A, Percentage of enriched diazotrophic OTUs in different soil profiles. B, Ternary plot depicting compartment specificity relative abundance of all enriched OTUs (> 5%) for diazotrophic distribution patterns in different soil depths. Each point corresponds with an OTU. Its position represents its relative abundance with regard to each compartment, and its size represents the average across all three compartments. Colored circles represent diazotrophic OTUs enriched in one compartment compared with the others (0–20, 20–40 and 0–60 cm). C, Aggregated relative abundance of each group of enriched OTUs in three soil depths are shown.

To better understand diazotrophic community distribution patter in the different soil profiles, we identified OTUs that were specifically enriched in various soil depths. Due to the fact that MNDS analysis on diazotrophic community structure did not show significant difference in different soil depths between CK and BC treatments (Fig 5), we performed analysis using combined samples from diazotrophic OTUs (Fig 7). Venn diagram analysis showed the similarity and overlap of species among various soil profile. Our results revealed that 59 (31.9%), 55 (29.7%) and 52 (28.1%) enriched diazotrophic OTUs were identified in 0–20, 20–40 and 40–60 cm soil depths, respectively. Thus indicating that diazotrophic OTUs community in the topsoil (0–20 cm) were higher compared to 20–40 and 40–60 cm soil depths (Fig 7A). Next, we characterized enriched diazotrophic OTUs across the three soil depths, ternary plot analysis was employed to identify enriched diazotrophic OTUs using the combined samples. Enriched diazotrophic OTUs were identified in different soil depths (Fig 7B). Unsurprisingly, subsequent boxplot analysis further revealed that these identified diazotrophic OTUs significantly depleted (*p* < 0.05) with soil depth (Fig 7C).

### 3.9 Multivariate ANOVA for the effects of soil depth, and different fertilizers on nifH OTUs, diversity, species richness, coverage and soil enzyme activities

Multivariate ANOVA analysis further revealed that soil depth significantly (p < 0.05) impacted diazotrophic OUTs, alpha diversity index (Shannon), species richness indice (Chao1), coverage, as well as soil enzyme activities, namely urease, cellulase, glucosidase and acid phosphatase compared to fertilization (Table 1). Furthermore, soil biochemical properties were significantly (*p* < 0.05) affected by soil depth than the different fertilization used (Table 2).

**Table 1:** Multivariate ANOVA for the effects of soil depth, and different fertilizers on nifH OTUs, diversity, species richness, coverage and soil enzyme activities.

	OUT	Shannon	Chao1	Coverage	Urease	Cellulase	Glucosidase	Acid phosphatase
Treatment	NS	NS	NS	NS	NS	NS	NS	NS
Depth	***	**	***	***	***	**	***	***
T X D	***	**	**	***	***	**	***	***

Depth stands for soil depth with 0–20 cm, 20–40 cm and 40–60 cm soil layers, treatment stands for fertilizer different fertilization with CK and BC.

**Table 2:** Multivariate ANOVA for the effects of soil depth, and different fertilizers on soil biochemical properties.

	pH	EC	TN	TC	TC/TN	AP	OM	AK	NO_3_^−^	NH_4_^+^
Treatment	NS	NS	NS	NS	**	NS	NS	NS	NS	NS
Depth	***	***	***	***	NS	***	NS	***	***	***
T X D	***	***	***	***	**	***	*	***	***	***

Depth stands for soil depth with 0–20 cm, 20–40 cm and 40–60 cm soil layers, treatment stands for fertilizer different fertilization with CK and BC.

### 3.10 Correlation between bacterial composition and soil properties

**Fig 8:**
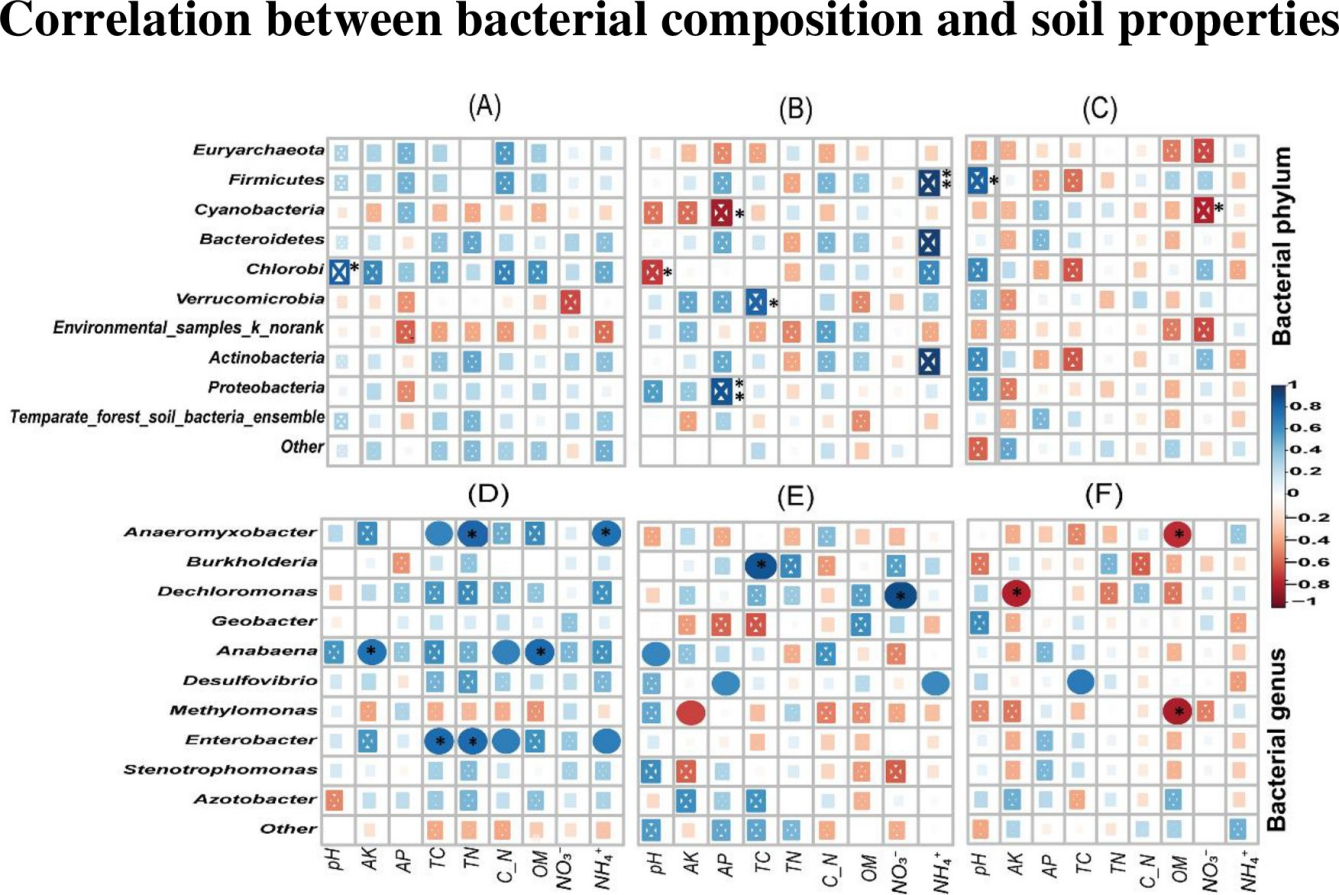
Pearson’s correlation coefficients for soil physio-chemical properties and the most abundant diazotrophic in different soil depth. Diazotrophic phyla in 0–20 cm (A), 20–40 cm (B) and 40–60 cm (C) and diazotrophic genera in 0–20 cm (D), 20–40 cm (E) and 40–60 cm (F) soil depths. The heatmap cells marked by “*” or “**” are statistically significant: * *p* < 0.05 and ** *p* < 0.01.

Pearson’s correlation coefficients were calculated to investigate the relationship between soil physio-chemical properties and the most abundant diazotrophic phyla and genera in three different soil depths. Our findings showed that diazotrophic phyla were to a lesser extent significantly and positively or negatively related to soil physiochemical properties compared to diazotrophic genera across the entire soil depth gradient (Fig 8A–8C). Soil pH was significantly and positively associated with the relative abundance of *Chlorobi* at soil depth 0–20 cm (Fig 8A). Furthermore, phyla *Firmicutes* and *Proteobacteria* showed a very strong and positive relationship with soil NH₄^+^ and AP at soil depths 20–40 cm, respectively. Whereas soil TC and *Verrucomicrobia* were positively and significantly correlated in soil depth 20–40 cm. However, *Cyanobacteria* and *Chlorobi* were significantly and negatively correlated with soil AP and pH at soil depth 20–40 cm, respectively (Fig 8B). At soil depth 40–60 cm, phylum *Firmicutes* revealed a strong and positive relationship with soil pH. However, NO_3_^−^ was significantly and negatively related to phylum *Cyanobacteria* (Fig 8C).

Meanwhile, our analysis revealed that more diazotrophic genera were to greater extent significantly and positively associated with many soil environmental variables especially in the surface soil 0–20 cm than diazotrophic phyla (Fig 8D–8F). *Anaeromyxobacter*, *Anabaena*, *Enterobacter* genera were significantly and positively associated with soil TC, NH₄^+^ and AK, OM, TC and TN in soil surface soil 0–20 cm, respectively (Fig 8D). Furthermore, in 20–40 cm soil layer, we observed that *Burkholderia* and *Dechloromonas* showed a strong and positive relationship with soil TC and NO_3_^−^, respectively (Fig 8E). However, in 40–60 cm soil depth, *Anaeromyxobacter*, *Methylomonas* and *Dechloromonas* were significantly and negatively correlated with soil OM and AK, respectively (Fig 8F).

## 4. Discussion

Biochar amended soil significantly increased the stalk weight and height of sugarcane. Similarly, Khalid et al. [43], Qayyum et al. [44] and Tian et al. [45] mentioned that BC application significantly improved crop yields. Soil depth is an important environmental gradient [46–48], as well as soil amendmet practices affecting soil physio-chemical properties [49–52]. In the current study, soil OM, TC, NH_4_^+^ NO_3_^−^, and AK significantly decreased with soil depth. Biochar is a promising alternative to improving soil fertility owing to the pivotal role it plays in building up reliable carbon storage [12,50]. Studies have revealed that BC amendment had profound effect on soil environmental variable, especially N and C cycles [53]. Similarly, our study revealed that biochar amended soil significantly increased soil OM, TC, NH_4_^+^ NO_3_^−^, and AK in one soil depth 0–20 cm compared to CK treatment, which we believed were resposible for the improved growth of sugarcane plant.

Soil enzyme activities are considered important indicators of fertile soil due to their important role they carry out in biochemical reaction and sustaining soil fertility [54,55]. In our previous study, enzymes associated with C and N cycles were enhanced in the topsoil than in subsoil [56]. In the current study, numerous patterns in the activity of soil enzymes were observed. Soil cellulase activity decreased with soil depth, while β-Glucosidase activity and acid phosphatase activity were significantly enhanced in 0–20 cm than 20–60 cm soil profile in both CK and BC treatments. On the other hand, the application of BC may harm soil enzyme activities by impeding or enhancing soil organic matter contents [57]. In the present study, cellulase and β-glucosidase activities in BC treatments revealed no significant change in the entire soil profile compared to CK. Biochar treatment significantly reduced urease activity and acid phosphatase activity in soil layer (0–20, 40–60 cm) and (0–20, 20–40 and 40–60 cm), respectively than CK. The diminishing trend of these enzymes could be attributed to the application of biochar.

Chu et al. [58] revealed that soil microbe community structure is naturally sensitive to environmental fluctuations, and is an important indicator of fertile soil. Diazotrophic abundance decrease with soil depths. Reardon et al. [25] revealed that diazotrophic abundance reduced in subsoil (10–20 cm) than upper soil layers (5–10 and 0–5 cm). Diazotrophic relative abundance was assessed at the phyla and genera levels to measure the impact of soil depth. We observed that biochar significantly influenced diazotrophic genera in 0–20 cm soil layer, but did not affect the diazotrophic phyla.

The utilization of biochar to amend soil can have a significant influence on the biotic and abiotic components of soil, thus significantly altering soil microbial abundance and community composition [14,59]. In the current study, BC amendment had no significant influence on diazotrophic abundance at the phyla level. However, compared to CK treatment, BC amended soil had profound impact on diazotrophic abundance at the genera level.

*Geobacter* was highly abundant in both BC and CK treatments. However, genus *Geobacter* was improved in one soil depth (20–40) in BC compared to CK treatment. This finding is roughly in consonant with studies conducted by Liao, Yaying, and Yao [60] and Liu et al. [14]. They reported that in biochar amended soil, *Geobacter* was among the dominant bacteria. Our previous study also revealed that *Geobacter* in the subsoil was enhanced compared to the topsoil [56]. This result further validates that *Geobacter* tends to grow in an anaerobic environment, which sequentially improves the lower soil with *Deltaproteobacteria* genera.

Information on free-living bacteria in the bulk soil is rare due to the diverse nature of soil microbe community structure. Besides, it is difficult to associate N fixation activities to each genus of diazotrophic genera [61]. However, Brown et al. [62] mentioned that *Anabaena* genus is known as a nitrogen fixer of a filamentous cyanobacteria genera. Chen et al. [63] reported that biochar amended soil increased soil microbial relative abundance at soil depth 0–15 cm. Similarly, the relative abundance of *Anabaena* was significantly high in one soil layer (0–20 cm) in BC amended soil compared to CK treatment.

*Azotobacter* is a nitrogen fixation bacteria that can stimulate soil rhizosphere microorganisms, protects plant against phytopathogen and boosts nutrient absorption which eventually enhance biological N fixation. In the current study, *Azotobacter* was significantly improved in soil profile 0–20 cm. Our result agreed with Eilers et al. [20] who reported that *Azotobacter* abundance is depth-dependent and was notably high in the rhizosphere soil.

*Burkholderia* is Gram-negative bacteria mainly composed of various soil-dwelling bacteria that exhibit different environmental functions, namely fixing nitrogen mutualists, pathogen and saprophyte [61,64]. Its distribution pattern in the soil is not clearly understood [64]. However, in three different agriculture management regimes, namely crop rotation, maize monoculture, and grassland. It was established that the area under maize monoculture and grassland cultivation was dominated by *Burkholderia* strains. In the current study, *Burkholderia* relative abundance was improved in the topsoil (0–20 cm) in BC amended soil compared to CK treatment. This finding partially agreed with Lin et al. [59], in which it was documented that *Burkholderia* relative abundance significantly increased under organic fertilizers.

*Stenotrophomonas* genus has been classified as a disease suppression bacteria and a plant growth promoter and a biocontrol agent [65]. A study found that *Stenotrophomonas* was significantly higher under no-mulching treatment at different soil depths. Similarly, our result revealed that *Stenotrophomona’s* relative abundance was higher at one soil depth (20–40 cm) in BC amended soil. It was evident in the current study where sugarcane growth parameters were enhanced significantly.

A nonmetric multidimensional scaling analysis was conducted to assess the impact of CK and BC treatments on diazotrophic community in different soil depths. The analysis revealed no significant difference in different soil depths between CK and BC treatments. However, using the combine samples, Venn diagram analysis clearly established that diazotrophs are depth-dependent. While ternary plot was able to distinctly identify enriched diazotrophic OTUs across the different soil profiles. Boxplot analysis further clearly exhibited significant difference in enriched OUTs among the soil depths. Our finding corroborated with the study conducted by Wang et al. [66] and Zgadzaj et al. [67]. They reported that bacteria community varied significantly with soil depth.

Furthermore, multivariate ANOVA analysis further confirmed that soil depth is one of the important environment gradients that greatly influence soil bacteria and soil biochemical properties [68]. We observed that soil depth significantly influenced diazotrophic OUTs, species richness indice, alpha diversity index, coverage, nifH gene copies, as well as soil enzyme activities, namely urease, cellulase, glucosidase and phosphatase and soil biochemical properties rather than fertilization. These results conformed with the findings of Fischer et al. [69] and Zhang et al. [56], in which they reported that soil bacterial, soil enzyme activities and soil physiochemical properties were depth dependent.

Soil environmental variables play an important role in influencing bacterial community structures [2,70]. Likewise, diazotrophic community structures are very responsive to soil environmental variables [71,72]. For instance, Zhang et al. [56] documented that soil pH and AK were the main factors affecting bacterial community compositions in the topsoil compared to AP and TC/TN. While in the subsoil, soil pH, AK and TC were the main factors in terms of changing bacterial community compositions. Similarly, redundancy analysis (RDA) revealed that diazotrophic community structures were very sensitive to soil environment, namely, TC, TN, OM, NH_4_^+^ and AP. Soil TC was the major impact factor, whereas soil TN, OM and NH_4_^+^ were the minor impact factor that caused a shift in diazotrophic community structures in soil depth 0–20 cm. Whereas at soil depths 20–40 and 40–60 cm, soil AP was the main driver shifting diazotrophic community structures.

## 5. Conclusions

This study explored the distribution patterns of diazotrophic genera and phyla, soil enzyme activities and soil physio-chemical properties in different soil depths under biochar amendments. We also assessed the response of sugarcane to these different soil management practices. Our findings revealed that soil depth had profound impact on the soil parameters measured. We also observed that diazotrophic relative abundance at the genera level, and enriched diazotrophic OTUs were significantly improved. These results are likely to enhance our understanding of how diazotrophic bacteria response to different soil management practices, as well as and their relationship with soil physio-chemical properties in different soil profiles.

## Supporting information

S1 Fig Dominant N2 fixers phyla trend in different soil profiles under different soil amendments. (DOCX)

S2 Fig Dominant N2 fixers genera trend in different soil profiles under different soil amendments. (DOCX)

S1 Table Diazotrophic α-diversity of indices containing diversity (Simpson) and species richness (Chao1). (DOCX)

S2 Table Diazotrophic OUTs and coverage in different soil depths under various amendments. (DOCX)

S1 File (RAR)
