# Supplementary material for: Depth-dependent influence of biochar application on the abundance and community structure of diazotrophic under sugarcane growth
Source: PLoS One. 2021 Jul 19;16(7):e0253970. doi: 10.1371/journal.pone.0253970 (PMC8289083; doi:10.1371/journal.pone.0253970)
Supplement: S2 Fig — (DOCX) [file pone.0253970.s002.docx]

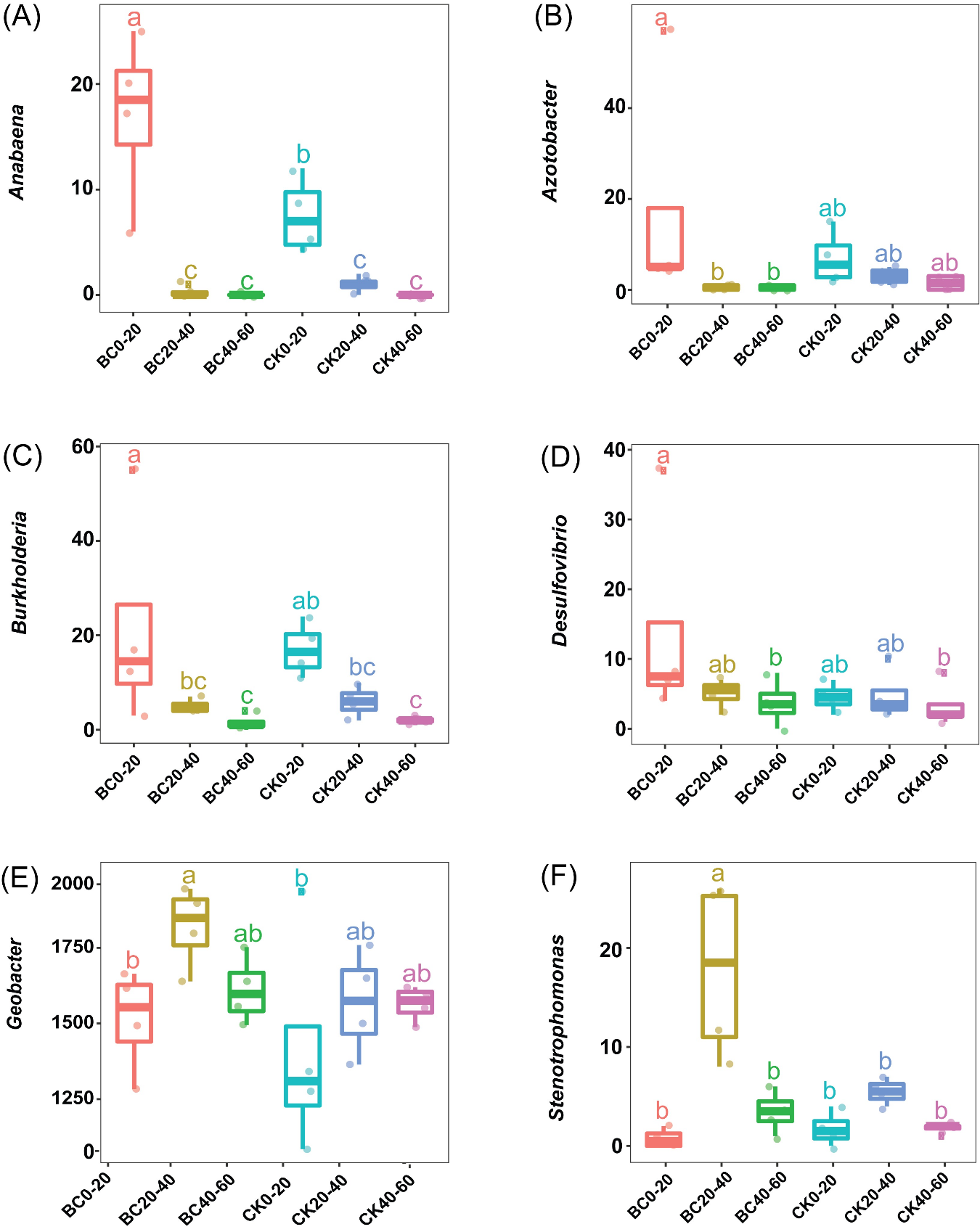


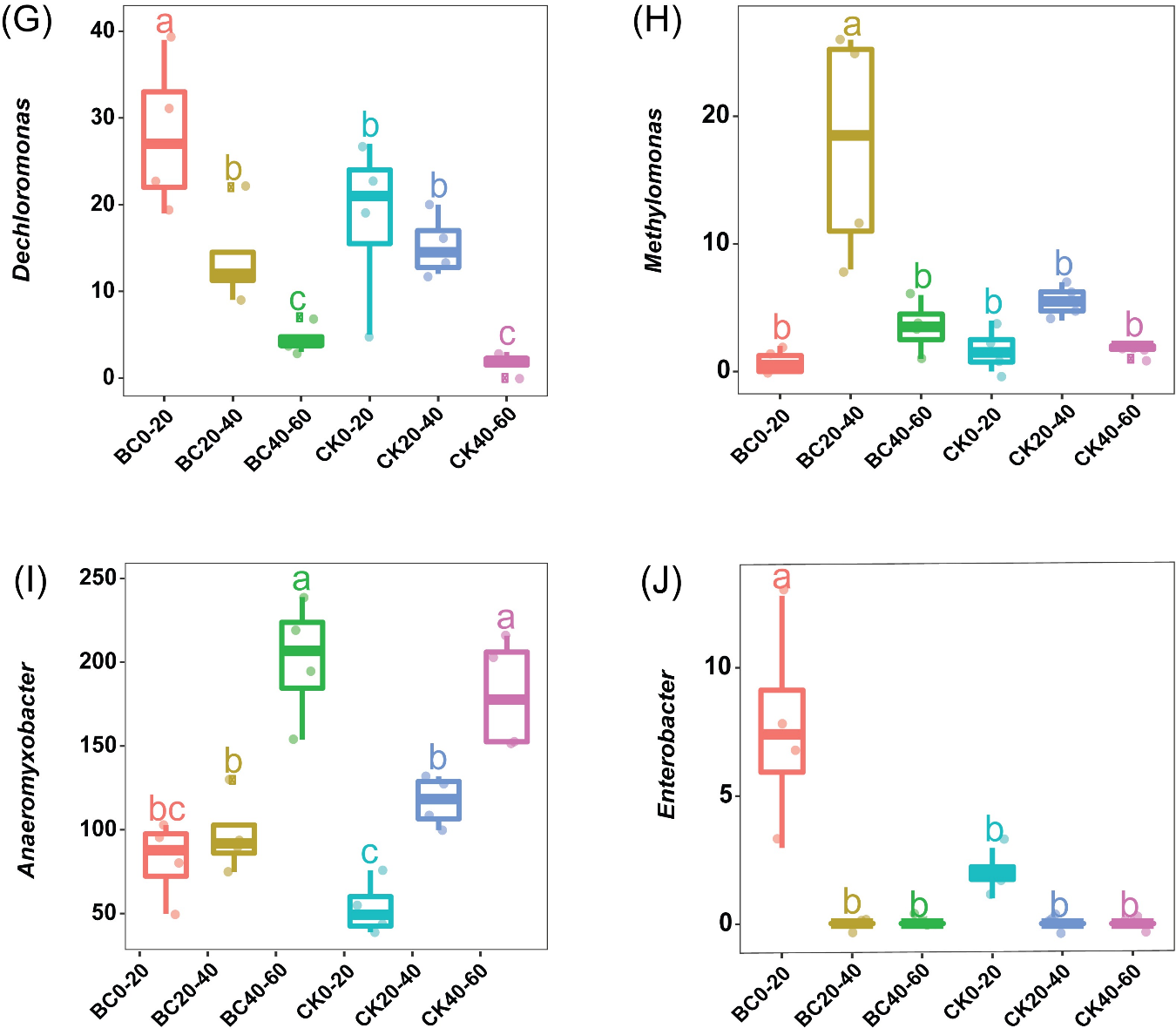


Fig S2. Dominant N2 fixers genera trend in different soil depth under different soil improvement measure. Different lowercase letters depict significant differences between treatments (Tukey test, *p* < 0.05).
