# Supplementary material for: Depth-dependent influence of biochar application on the abundance and community structure of diazotrophic under sugarcane growth
Source: PLoS One. 2021 Jul 19;16(7):e0253970. doi: 10.1371/journal.pone.0253970 (PMC8289083; doi:10.1371/journal.pone.0253970)
Supplement: S1 Table — (DOCX) [file pone.0253970.s003.docx]

**Table S1**. **Diazotrophic α-Diversity of indices containing diversity (Simpson) and species richness (Chao1)**

**Diversity Richness**

| **Treatments** |  | **Soil depth(cm)** | | **Simpson- index** | **Chao1- index** |
| --- | --- | --- | --- | --- | --- |
| CK |  | 0-20 |  | 0.03±0.02a | 1569.58±133.90d |
| CK |  | 20-40 |  | 0.01±0.00a | 2259.81±181.00bcd |
| CK |  | 40-60 |  | 0.02±0.00a | 2011.48±60.53abcd |
| BC |  | 0-20 |  | 0.01±0.00a | 1778.83±68.90bcd |
| BC |  | 20-40 |  | 0.01±0.00a | 2160.74±32.04bcd |
| BC |  | 40-60 |  | 0.02±0.00a | 2032.94±67.83bcd |

Diazotrophic α-Diversity of indices containing of diversity (Simpson) and species richness (Chao1) under BC, biochar and CK, control in different soil layers (0-20, 20-40 and 40-60 cm). Data are means ± standard deviation (n=4). Different letters above columns indicate significant difference among fertilizer treatments (*p* < 0.05).
