# Supplementary material for: Depth-dependent influence of biochar application on the abundance and community structure of diazotrophic under sugarcane growth
Source: PLoS One. 2021 Jul 19;16(7):e0253970. doi: 10.1371/journal.pone.0253970 (PMC8289083; doi:10.1371/journal.pone.0253970)
Supplement: S2 Table — (DOCX) [file pone.0253970.s004.docx]

**Table S2**. **Diazotrophic OUTs and Coverage in different soil depths under various amendments**

| **Treatments** |  | **Soil depth(cm)** |  | **OUTs** |  | **Coverage** |
| --- | --- | --- | --- | --- | --- | --- |
| CK |  | 0-20 |  | 1028.00±7.80bcd |  | 0.91±0.01a |
| CK |  | 20-40 |  | 1040.50±27.04bcd |  | 0.87±0.01e |
| CK |  | 40-60 |  | 1024.00±22.22bcd |  | 0.88±0.00bcde |
| BC |  | 0-20 |  | 1115.50±18.79abc |  | 0.90±0.00abc |
| BC |  | 20-40 |  | 1249.25±14.35a |  | 0.87±0.00de |
| BC |  | 40-60 |  | 1189.00±15.26ab |  | 0.88±0.00cde |

Diazotrophic enriched OUTs and coverage under BC, biochar and CK, control in different soil layers (0-20, 20-40 and 40-60 cm). Data are means ± standard deviation (n=4). Different letters above columns indicate a significant difference among fertilizer treatments *(p* < 0.05).
